# Real-world pharmacovigilance and molecular mechanisms of fruquintinib: SRC and STAT3 as potential off-target mediators of proteinuria

**DOI:** 10.3389/fphar.2026.1796154

**Published:** 2026-03-13

**Authors:** Duogang Xu, Zhicheng Dai, Yulei He, Pengcheng Zhao, Shucheng Lin, Xiaofang Chen, Zichun Wei, Jianfa Rao, Jing Zhou, Youtao Guan, Wenwen Huang, Songlin Yang, Jing Tan

**Affiliations:** 1 Department of General Surgery, Yan’an Hospital Affiliated to Kunming Medical University, Kunming, China; 2 Key Laboratory of Tumor Immunological Prevention and Treatment of Yunnan Province, Kunming, China; 3 Trauma Center, Shanghai General Hospital, Shanghai Jiao Tong University School of Medicine, Shanghai, China; 4 Department of Chinese Integrative Medicine Oncology, the First Affiliated Hospital of Anhui Medical University, Hefei, China; 5 Department of Urology, Yan’an Hospital Affiliated to Kunming Medical University, Kunming, China; 6 Central Laboratory of Yan’an Hospital Affiliated to Kunming Medical University, Kunming, China; 7 Department of Pathology, Yan’an Hospital Affiliated to Kunming Medical University, Kunming, China

**Keywords:** adverse drug events, FAERS database, fruquintinib, metastatic colorectal cancer, network pharmacology, pharmacovigilance

## Abstract

**Background:**

Fruquintinib, a highly selective inhibitor of vascular endothelial growth factor receptors (VEGFR) 1–3, has demonstrated significant survival benefits in metastatic colorectal cancer (mCRC). While pivotal clinical trials have characterized its general toxicity profile, the molecular mechanisms driving specific, high-risk adverse events in the real-world setting remain underexplored.

**Methods:**

We adopted a “bedside-to-bench” reverse translational strategy to systematically evaluate the safety profile of Fruquintinib. First, we analyzed spontaneous reports from the FDA Adverse Event Reporting System (FAERS) (Q4 2023–Q1 2025) using disproportionality algorithms (ROR, PRR, BCPNN, and MGPS) to detect significant safety signals. Recognizing proteinuria as a clinically critical organ-specific toxicity, we subsequently prioritized it for mechanistic investigation. A “drug-target-disease” network was constructed, followed by molecular docking and 100-nanosecond (ns) molecular dynamics (MD) simulations to elucidate the underlying molecular etiology.

**Results:**

A total of 1,632 Fruquintinib-related reports were identified, with 66.2% of adverse events occurring within the first 30 days of therapy. Significant disproportionality signals were detected for myelosuppression (ROR = 29.31, 95% CI: 23.40–36.71), dysphonia (ROR = 21.96, 95% CI: 18.37–26.26), and proteinuria (ROR = 16.92, 95% CI: 11.81–24.23). Network pharmacology analysis mapped the intersection of drug and disease targets, identifying SRC and STAT3 as core hub kinases. Molecular docking revealed that Fruquintinib exhibits favorable theoretical binding affinities to the active pockets of SRC (−7.8 kcal/mol) and STAT3 (−7.4 kcal/mol), surpassing its affinity for several canonical targets. MD simulations further confirmed the rigorous dynamic stability of these complexes (RMSD ∼0.22 nm) and revealed a distinct induced-fit binding mechanism.

**Conclusion:**

This study provides the first integration of pharmacovigilance data with biophysical modeling for Fruquintinib. Beyond corroborating established class related safety profiles, we hypothesize a novel dual hit molecular framework for renal toxicity. This clinical association might involve both classical vascular endothelial growth factor receptor blockade and the potential off target structural interactions of SRC and STAT3. These insights underscore the importance of early renal monitoring and offer structural insights into the drug’s safety profile.

## 1 Introduction

Colorectal cancer (CRC) remains one of the most prevalent and lethal malignancies worldwide, currently ranking third in incidence and second in mortality according to recent global cancer statistics (Sung et al., 2021). Although advances in both screening and therapeutic strategies have been achieved, approximately 25% of patients are still diagnosed at the metastatic stage, and nearly 60% will develop distant metastases within 5 years (Siegel et al., 2023). The prognosis for metastatic colorectal cancer (mCRC) is therefore poor, with the 5-year survival rate consistently reported at below 20% (Van Cutsem et al., 2016; Bens et al., 2018). At present, systemic chemotherapy constitutes the cornerstone of mCRC management, and it is frequently combined with targeted agents to improve survival outcomes (Fakih, 2015).

Fruquintinib is an oral, small-molecule tyrosine kinase inhibitor that selectively targets vascular endothelial growth factor receptors (VEGFR) 1, 2, and 3 (Sun et al., 2014). Through inhibition of VEGFR-mediated angiogenesis, Fruquintinib reduces tumor vascularization and consequently suppresses tumor growth (Patell et al., 2024; Chen and Jiang, 2019). The agent was first approved in China in 2018 and subsequently received approval in the United States in 2023 for use in mCRC patients previously treated with standard chemotherapy and targeted regimens. Evidence from pivotal clinical trials, including the FRESCO and FRESCO-2 studies, demonstrated significant improvements in both overall survival and progression-free survival among heavily pretreated mCRC populations (Dasari et al., 2023; Li et al., 2018). Nevertheless, consistent with other anti-angiogenic therapies, Fruquintinib is linked to a distinctive profile of adverse drug events (ADEs), most notably hypertension, proteinuria, hand–foot skin reaction, and hematologic toxicities (Dasari et al., 2023; Yonatan et al., 2025; Van Cutsem et al., 2012). Several of these ADEs can be severe, posing challenges for treatment adherence and potentially compromising patient outcomes.

The Food and Drug Administration Adverse Event Reporting System (FAERS) is a large, publicly accessible pharmacovigilance resource that compiles spontaneous reports of suspected ADEs submitted by healthcare professionals, consumers and manufacturers (Zhang et al., 2024). This database has become a cornerstone of post-marketing safety evaluation, particularly for newly approved agents or drugs with limited long-term safety data (Li et al., 2024). To identify potential safety signals, disproportionality analyses such as the reporting odds ratio (ROR) and proportional reporting ratio (PRR) are commonly applied, allowing detection of drug–event pairs that occur more frequently than expected (Evans et al., 2001; van Puijenbroek et al., 2002). FAERS-based investigations have previously delineated the safety profiles of numerous anticancer therapies, including VEGFR inhibitors, thereby offering real-world evidence that complements and extends findings from clinical trials (Miura et al., 2014; Kan et al., 2025).

Several VEGFR inhibitors with mechanisms comparable to Fruquintinib have already been evaluated using FAERS. Regorafenib, an oral multikinase inhibitor approved for refractory metastatic colorectal cancer, has been associated in FAERS analyses with hand–foot skin reaction, hepatotoxicity, and hemorrhagic events, and notably, renal disorders including proteinuria (Russo et al., 2023). Apatinib, a selective VEGFR-2 inhibitor commonly prescribed in China for gastric and hepatocellular carcinoma, has similarly demonstrated signals for hypertension, proteinuria, and hand–foot syndrome in post-marketing pharmacovigilance studies (Spini et al., 2022). Lenvatinib, which has gained global approval for hepatocellular and thyroid cancers, has shown prominent FAERS associations with hypertension, proteinuria, and thyroid dysfunction (Yu et al., 2024). Collectively, these observations underscore the utility of FAERS for identifying both class-related and drug-specific toxicities among VEGFR-targeted therapies. However, although proteinuria is widely considered a class effect of multi-kinase inhibitors and is often linked to on-target hemodynamic changes, the drug-specific mechanisms underlying direct renal cell injury remain incompletely defined. Given that fruquintinib was designed for high selectivity toward VEGFR1, 2and 3, it is important to determine whether its renal toxicity is primarily on-target or may also involve distinct, previously underappreciated off-target kinase modulation with potential clinical relevance. Addressing this mechanistic gap provides a clear rationale and underscores the novelty of our study.

Traditional pharmacovigilance studies primarily identify statistical signals but often fail to explain the underlying biological mechanisms. For instance, while clinical trials established proteinuria as a major toxicity, its real-world risk relative to other adverse events requires further quantification. Furthermore, the precise molecular drivers remain unclear. It is currently unknown whether Fruquintinib induces renal injury solely through on-target VEGFR inhibition or involves off-target modulation of other signaling pathways. Therefore, integrating clinical data mining with molecular investigation is necessary to understand the etiology of this adverse event and guide precise management.

Therefore, this study adopted a comprehensive bedside-to-bench reverse translational strategy. First, we utilized the FAERS database to systematically characterize the full spectrum of Fruquintinib-associated adverse events, identifying both novel signals (such as dysphonia) and confirming high-risk toxicities. Subsequently, recognizing proteinuria as the most prominent safety signal with a critically high reporting odds ratio (ROR = 16.92), we employed network pharmacology, molecular docking, and molecular dynamics (MD) simulations to investigate its underlying mechanism. This integrated approach allowed us to construct a “drug-target-disease” network and validate the binding stability of Fruquintinib with potential core targets (e.g., SRC, STAT3), providing novel molecular insights into the etiology of this clinically significant toxicity.

## 2 Methods

### 2.1 Data source

Data were retrieved from the FAERS Quarterly Data Files, covering the period from the fourth quarter of 2023 (Q4 2023) to the first quarter of 2025 (Q1 2025). This specific temporal window was deliberately selected to coincide with the official approval of Fruquintinib by the United States Food and Drug Administration in November 2023. Initiating data extraction from this exact regulatory milestone ensures the comprehensive capture of postmarketing safety reports immediately following the integration of the drug into broader clinical practice. FAERS comprises seven primary datasets: Demographic and Administrative Information (DEMO), Drug Information (DRUG), Adverse Event Reports (REAC), Patient Outcomes (OUTC), Indications for Use (INDI), Therapy Start and End Dates (THER), and Report Sources (RPSR). This study focused on reports containing the search terms “Fruquintinib,” “Fruzaqla,” or “Elunate,” including both monotherapy and combination regimens.

### 2.2 Data cleaning and processing

**FIGURE 1:**
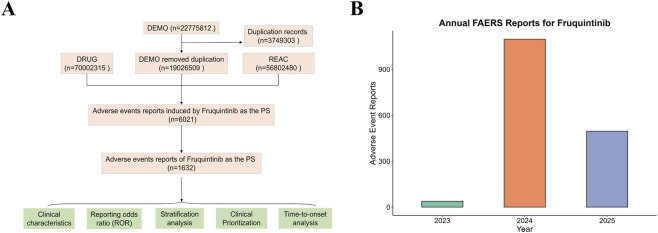
Data selection process and temporal trends of adverse event reports associated with Fruquintinib in the FAERS database. **(A)** Flowchart illustrating the data screening and analysis strategy. **(B)** Annual distribution of reported adverse events related to Fruquintinib from 2023 to 2025.

Potential duplicates were addressed according to FDA-recommended procedures by grouping records using CASEID and PRIMARYID and retaining only the entry with the latest FDA_DT. Reports with incomplete or ambiguous key variables, inconsistent data, or clear input errors were excluded. AEs were standardized using Preferred Terms (PTs) and System Organ Classes (SOCs) from the Medical Dictionary for Regulatory Activities (MedDRA®, version 28.0), maintained by the MedDRA Maintenance and Support Services Organization (MSSO) under the International Council for Harmonisation (ICH). The workflow for data acquisition, preprocessing, and analysis is summarized in Figure 1A. The study followed the Strengthening the Reporting of Observational Studies in Epidemiology (STROBE) guidelines.

### 2.3 Network pharmacology and functional enrichment analysis

To elucidate the molecular mechanisms underlying the key adverse event identified from FAERS, we performed a network pharmacology analysis. Leveraging the chemical structure of Fruquintinib, potential therapeutic targets were predicted using the SwissTargetPrediction and PharmMapper databases. To ensure the reliability of predicted targets, results were strictly restricted to *Homo sapiens*. Furthermore, only targets with a probability score greater than 0.5 in SwissTargetPrediction and ranked within the top 100 by fit score in PharmMapper were retained. Simultaneously, targets associated with specific adverse events were screened from the GeneCards and OMIM databases. To maintain stringent data quality for disease-related targets, only those with a correlation score above 2 were included in subsequent intersection analysis. The intersection of drug-related and disease-related targets was identified using a Venn diagram. A Protein-Protein Interaction (PPI) network was constructed using the STRING database (version 12.0) with a medium confidence score (>0.400). The resulting network was visualized in Cytoscape (version 3.10.1). To screen for core regulatory proteins, the CytoHubba plugin was employed to identify hub genes based on the Degree algorithm, where node color intensity was mapped to degree centrality. Functional enrichment analysis, including Gene Ontology (GO) and Kyoto Encyclopedia of Genes and Genomes (KEGG), was performed using the R package “clusterProfiler”. Adjusted P-values <0.05 were considered statistically significant.

### 2.4 Molecular docking verification

To validate the binding potential between Fruquintinib and the identified core targets, molecular docking simulations were performed. The 3D crystal structures of the priority targets, including EGFR, IGF1R, SRC, and STAT3, were retrieved from the RCSB Protein Data Bank (PDB). The chemical structure of Fruquintinib was obtained from the PubChem database and energy-minimized using the MM2 force field. Prior to docking, the protein structures were prepared by removing water molecules and co-crystallized ligands, followed by the addition of polar hydrogen atoms and Gasteiger charges using AutoDock Tools. To ensure comprehensive conformational sampling, the geometric center of the grid box was strictly anchored to the coordinates of the native cocrystallized ligands within each target protein. The grid dimensions were appropriately expanded to fully cover the active site and accommodate the spatial exploration of the ligand. Semiflexible docking was subsequently conducted using AutoDock Vina version 1.1.2 utilizing an exhaustiveness parameter of 8. The binding affinity (kcal/mol) was used as the primary metric, where values lower than −7.0 kcal/mol indicate strong binding stability. Visualization of the optimal binding conformations and hydrogen bond interactions was performed using PyMOL.

### 2.5 Molecular dynamics simulation

To further assess the temporal stability and conformational dynamics of the ligand-protein complexes under physiological conditions, we performed 100-nanosecond (ns) MD simulations using the GROMACS software package (version 2023). A production run of 100 nanoseconds was executed at 310 K and 1 atmospheric pressure. This simulation duration was specifically designated because it provides an adequate timescale to observe the structural relaxation of protein side chains and the stable equilibration of kinase and inhibitor complexes under physiological conditions. Furthermore, the structural convergence of the simulated systems was rigorously assessed by continuously monitoring the Root Mean Square Deviation trajectories over time. The attainment of a stable plateau in these trajectories confirmed that the protein and ligand complexes had successfully reached thermodynamic equilibrium without significant structural drift prior to the conclusion of the simulation. Based on the molecular docking results, the two complexes with the highest binding affinities—SRC-Fruquintinib and STAT3-Fruquintinib—were prioritized for dynamic simulation. The CHARMM36 force field was applied to generate protein topologies, while ligand parameters were derived using the CGenFF server. The systems were solvated in a cubic box using the TIP3P water model and neutralized by adding Na+ and Cl-ions (0.15 M). Following energy minimization (steepest descent algorithm) and equilibration (NVT and NPT ensembles for 100 ps each), a production run of 100 ns was executed at 310 K and 1 bar. Trajectory stability was quantified using Root Mean Square Deviation (RMSD), Root Mean Square Fluctuation (RMSF), Radius of Gyration (Rg), and Solvent Accessible Surface Area (SASA).

### 2.6 Statistical analysis

Signal detection was conducted using disproportionality analysis, a standard pharmacovigilance approach that assesses whether a given AE is reported more frequently for the drug of interest than for other drugs in the database. Four established algorithms were applied: ROR, PRR, Bayesian Confidence Propagation Neural Network (BCPNN), and Multi-Item Gamma Poisson Shrinker (MGPS). Adverse events (AEs) meeting the predefined thresholds for all four algorithms were considered positive signals. The 2 × 2 contingency framework and algorithmic formulas are provided in Tables 1, 2. All statistical analyses and data visualization were performed in R software (version 4.3.2) using appropriate analytical and plotting packages.

**TABLE 1:** Disproportionality analysis 2 × 2 contingency table for Fruquintinib-related adverse events in FAERS.

Drug exposure	Target AEs	Non-target AEs	Total
Fruquintinib	a	b	a + b
Non-fruquintinib	c	d	c + d
Total	a + c	b + d	N = a + b + c + d

AEs, adverse events.

**TABLE 2:** Signal detection algorithms applied in the disproportionality analysis of Fruquintinib-related adverse events, with equations and criteria.

Algorithms	Equation	Criteria
ROR	ROR = (a/c)/(b/d) 95% CI = e^(ln (ROR) ± 1.96√(1/a + 1/b + 1/c + 1/d))	95% CI (lower limit) > 1, a ≥3
PRR	PRR = [a/(a + b)]/[c/(c + d)] 95% CI = e^(ln (PRR) ± 1.96√(1/a −1/(a + b) + 1/c − 1/(c + d)))	95% CI (lower limit) > 1, a ≥3
BCPNN	IC = log_2_ ((a + b + c + d)/((a + c) (a + b))) IC25 = e^(ln (IC) − 1.96√(1/a + 1/b + 1/c + 1/d))	IC25 > 0, a ≥3
MGPS	EBGM = (a + b + c + d)/((a + c) (a + b)) EBGM05 = e^(ln (EBGM) − 1.64√(1/a + 1/b + 1/c + 1/d))	EBGM05 > 2, a >0

ROR, reporting odds ratio; CI, confidence interval; PRR, proportional reporting ratio; BCPNN, bayesian confidence propagation neural network; IC, information component; IC025, the lower limit of the 95% CI, of the IC; MGPS, multiple gamma Poisson shrinkage; EBGM, empirical Bayesian geometric mean; EBGM05, empirical Bayesian geometric mean lower 95% CI, for the posterior distribution.

## 3 Results

### 3.1 Patient characteristics and reporting trends

A total of 1,632 AE reports associated with Fruquintinib were identified in the FAERS database. The number of reports was minimal in 2023, reflecting the drug’s U.S. approval in November of that year, peaked in 2024, and decreased in early 2025 due to the data collection ending in the first quarter (Figure 1B). Males represented a slightly higher proportion of cases (51.9%) than females (41.1%). Among reports with available weight data, the majority of patients weighed between 50 and 100 kg (14.6%), followed by <50 kg (2.8%) and >100 kg (1.5%). Regarding age, the largest proportions were in the 18–64.9 years group (24.4%) and the 65–85 years group (19.6%), with smaller proportions in patients aged <18 years (5.6%) and >85 years (0.7%). This age distribution is consistent with the clinical use of Fruquintinib, which is primarily indicated for the treatment of mCRC, a condition more prevalent among middle-aged and older adults. In terms of clinical outcomes, death was reported in 33.9% of cases and hospitalization occurred in 24.8%, while life-threatening events (1.3%) and disability (0.6%) were less frequent. The relatively high proportion of severe outcomes may, in part, reflect the advanced disease stage in patients receiving Fruquintinib for mCRC. Consumers were the primary reporters (34.0%), followed by physicians (22.1%), other health professionals (12.7%), and pharmacists (6.4%). Geographically, the United States contributed the majority of reports (62.9%), followed by China (14.6%) and Japan (8.1%), with smaller proportions from other countries (Table 3).

**TABLE 3:** Demographic and clinical characteristics of patients with Fruquintinib-associated adverse event reports in FAERS.

Factors	Number of reports (%)
Overall	1632 (100%)
Gender	
Female	671 (41.1%)
Male	847 (51.9%)
Missing	114 (7.0%)
Weight (kg)	
<50	45 (2.8%)
50-100	239 (14.6%)
>100	25 (1.5%)
Missing	1,323 (81.1%)
Age (years)	
<18	92 (5.6%)
>85	12 (0.7%)
18-64.9	399 (24.4%)
65-85	320 (19.6%)
Missing	809 (49.6%)
Outcome	
Death	554 (33.9%)
Disability	9 (0.6%)
Hospitalization	405 (24.8%)
Life-threatening	21 (1.3%)
Other	643 (39.4%)
Reporters	
Consumer	555 (34.0%)
Health professional	208 (12.7%)
Pharmacist	105 (6.4%)
Physician	360 (22.1%)
Missing	404 (24.8%)
Reported countries	
United States	1,027 (62.9%)
China	238 (14.6%)
Japan	132 (8.1%)
United Kingdom	49 (3.0%)
France	41 (2.5%)
Italy	34 (2.1%)

### 3.2 SOC distribution and signal detection

**FIGURE 2:**
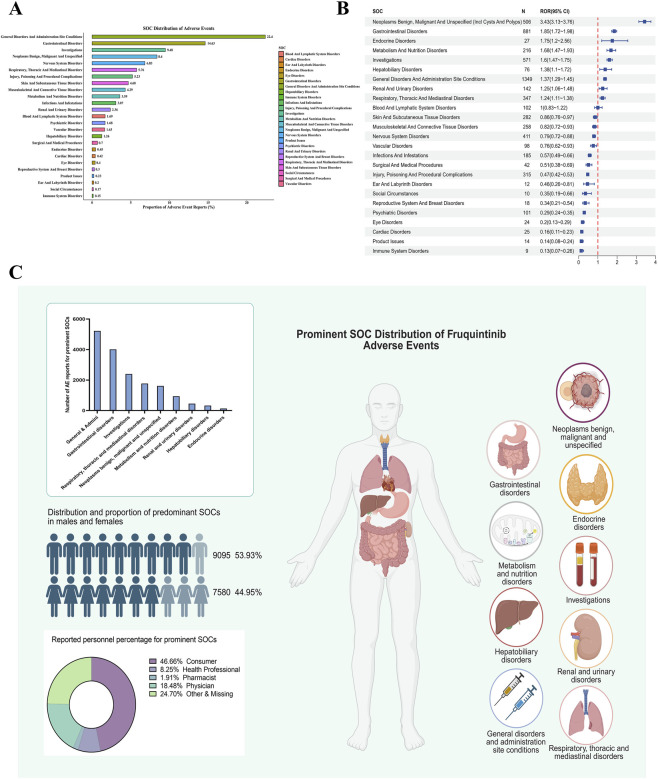
Signal detection and analysis of adverse events by System Organ Class (SOC) associated with Fruquintinib. **(A)** Distribution of adverse event reports classified by MedDRA SOC terms. Each bar represents the percentage of total reports attributed to a specific SOC category. **(B)** Forest plot of Reporting Odds Ratios (ROR) with 95% confidence intervals (CI) for SOC-specific adverse events. The vertical line represents the threshold for signal detection. **(C)** Characteristics of prominent SOCs that exhibited statistically significant RORs.

AEs related to Fruquintinib were categorized into 25 SOCs (Figure 2A). In terms of reporting frequency, General disorders and administration site conditions accounted for the largest share (22.4%), followed by Gastrointestinal disorders (14.63%) and Investigations (9.48%). Other SOCs with notable proportions included Neoplasms benign, malignant and unspecified (including cysts and polyps) (8.4%) and Nervous system disorders (6.83%). Respiratory, thoracic and mediastinal disorders (5.76%) and Injury, poisoning and procedural complications (5.23%) also ranked among the higher categories, indicating that AEs spanned multiple physiological systems rather than being confined to a single organ class.

Signal detection analysis revealed several SOCs with a ROR significantly greater than 1 (Figure 2B). The strongest disproportionality was observed for Neoplasms benign, malignant and unspecified (ROR = 3.43, 95% CI: 3.13–3.76), followed by Gastrointestinal disorders (ROR = 1.85, 95% CI: 1.72–1.98) and Endocrine disorders (ROR = 1.75, 95% CI: 1.20–2.56). Elevated signals were also noted for Metabolism and nutrition disorders (ROR = 1.68, 95% CI: 1.47–1.93), Investigations (ROR = 1.60, 95% CI: 1.47–1.75), Hepatobiliary disorders (ROR = 1.38, 95% CI: 1.10–1.72), General disorders and administration site conditions (ROR = 1.37, 95% CI: 1.29–1.45), Renal and urinary disorders (ROR = 1.25, 95% CI: 1.06–1.48), and Respiratory, thoracic and mediastinal disorders (ROR = 1.24, 95% CI: 1.11–1.38). These findings indicate that FAAEs encompass multiple organ systems (Figure 2C), with particularly strong reporting disproportionality observed in Neoplasms benign, malignant and unspecified (including cysts and polyps), Gastrointestinal disorders, Endocrine disorders, Metabolism and nutrition disorders, and Investigations. The elevated ROR for these categories suggests that, beyond the general distribution of AEs, Fruquintinib use is more frequently associated with these specific SOCs, reflecting both the drug’s pharmacological profile and the clinical characteristics of the treated population.

### 3.3 Preferred Term (PT) level signal detection

**FIGURE 3:**
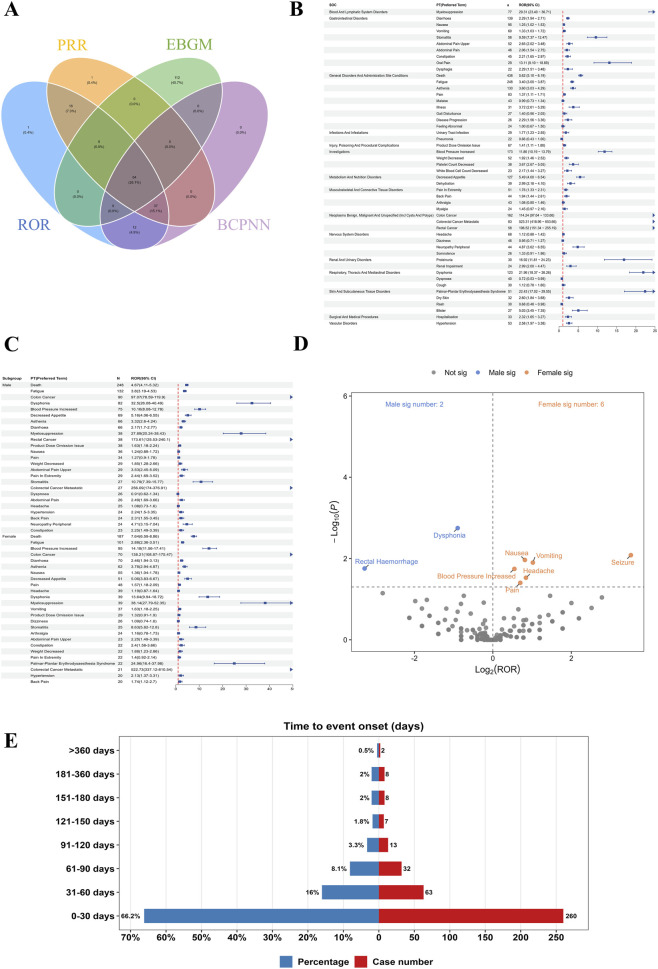
Comprehensive analysis of specific adverse event signals: detection overlap, risk quantification, sex disparities, and onset timing. **(A)** Venn diagram showing the overlap of significant signals identified by four different signal detection algorithms. **(B)** Forest plot of Reporting Odds Ratios (ROR) with 95% confidence intervals (CI) for adverse events at the Preferred Term (PT) level. **(C)** Forest plots displaying sex-specific RORs, comparing the risk of specific adverse events between male and female subgroups. **(D)** Volcano plot illustrating the statistical significance of sex differences in adverse event signals. The x-axis represents the difference in risk (Log2 Fold Change/ROR ratio), and the y-axis represents the significance level (-Log10 P-value). **(E)** Time-to-onset distribution of adverse events. The red bars indicate the number of cases, while the blue bars represent the percentage of total reports within each time interval.

At the PT level, signal detection revealed a broad range of FAAEs across multiple SOCs. As depicted in the Venn diagram (Figure 3A), 64 PTs (26.1%) were identified as positive signals by all four disproportionality algorithms (ROR, PRR, EBGM, and BCPNN), representing the most robust and consistent safety signals. Additionally, 37 PTs (15.1%) were concurrently detected by ROR, PRR, and BCPNN, whereas 112 PTs (45.7%) were uniquely detected by EBGM. Smaller subsets were detected exclusively by a single method or by other combinations of algorithms (Supplementary Table S1).

In addition, multiple FAAEs demonstrated significant disproportionality signals. The highest RORs were observed for myelosuppression (ROR = 29.31, 95% CI: 23.40–36.71), palmar–plantar erythrodysaesthesia syndrome (ROR = 22.43, 95% CI: 17.02–29.55), dysphonia (ROR = 21.96, 95% CI: 18.37–26.26), proteinuria (ROR = 16.92, 95% CI: 11.81–24.23), and blood pressure increased (ROR = 11.86, 95% CI: 10.19–13.79). Several events aligned with the known toxicity spectrum of VEGFR inhibitors, including hypertension-related terms, proteinuria, and dermatologic toxicity. Gastrointestinal disorders such as diarrhea (ROR = 2.29, 95% CI: 1.94–2.71), abdominal pain (ROR = 2.65, 95% CI: 2.02–3.48), and stomatitis (ROR = 9.59, 95% CI: 7.37–12.47) also exhibited notable signals. Additionally, urinary tract infection (ROR = 1.77, 95% CI: 1.23–2.55) was detected, suggesting a potential predisposition to bacterial infections during treatment. Platelet count decreased (ROR = 3.67, 95% CI: 2.67–5.05) was also identified, which may reflect an increased risk of bleeding (Figure 3B). The pattern of prominent AEs highlights potential bleeding risk, urinary system involvement, dermatologic toxicity, and gastrointestinal complications, underscoring both class-related and potentially Fruquintinib-specific safety concerns in mCRC patients.

### 3.4 PT analysis at the subgroup level

We first conducted an analysis in the gender subgroup (Figure 3C). As expected for an agent indicated in mCRC, gastrointestinal malignancy–related PTs exhibited extremely high RORs in both sexes. Beyond these indications, in male patients, notable disproportionality signals were observed for Dysphonia (ROR = 32.52, 95% CI = 26.80–40.49), Myelosuppression (ROR = 27.89, 95% CI = 20.24–38.43), and Stomatitis (ROR = 10.79, 95% CI = 7.39–15.77). In female patients, strong signals included Myelosuppression (ROR = 38.14, 95% CI = 27.79–52.35), Palmar–plantar erythrodysaesthesia syndrome (ROR = 24.96, 95% CI = 16.47–37.98), Blood pressure increased (ROR = 14.18, 95% CI = 11.56–17.41), and Stomatitis (ROR = 8.63, 95% CI = 5.82–12.80). Palmar–plantar erythrodysaesthesia syndrome demonstrated a pronounced sex-specific disparity, with a markedly stronger signal in females compared to males. Furthermore, the ROR for blood pressure increased was substantially higher in females, accompanied by elevated signals for other dermatologic AEs, suggesting a potential sex-related predisposition to vascular and cutaneous toxicities.

Moreover, we conducted a gender difference analysis and plotted a volcano plot (Figure 3D), where the orange and blue dots represent the high-risk ADEs in females and males, respectively. In female patients, significant ADEs included nausea, vomiting, headache, pain, blood pressure increased, and seizure. In male patients, significant ADEs were dysphonia and rectal haemorrhage. These findings indicate a broader spectrum of significant ADEs in females compared to males.

In the age subgroup analysis, the pattern of prominent ADEs was generally similar between adults and older patients. In the reporter type analysis, health professional reports most often involved dysphonia, myelosuppression, stomatitis, and blood pressure increased, whereas consumer reports more frequently included palmar–plantar erythrodysaesthesia syndrome, proteinuria, hypertension, and dysphonia (Supplementary Figures S1-S2).

### 3.5 Time to onset of AEs associated with fruquintinib

A total of 393 AEs reports contained detailed onset time information. As illustrated in Figure 3E, the majority of AEs (66.2%) occurred within the first 30 days after initiation of Fruquintinib, followed by 16.0% between 31 and 60 days and 8.1% between 61 and 90 days. The incidence markedly decreased thereafter, with less than 3.5% of events reported beyond 90 days. Notably, only 0.5% of AEs were observed more than 1 year after treatment initiation. These findings highlight that the early phase of therapy represents the period of highest AE risk, underscoring the importance of close monitoring during the initial month of Fruquintinib treatment.

### 3.6 Network pharmacology analysis of fruquintinib associated proteinuria

**FIGURE 4:**
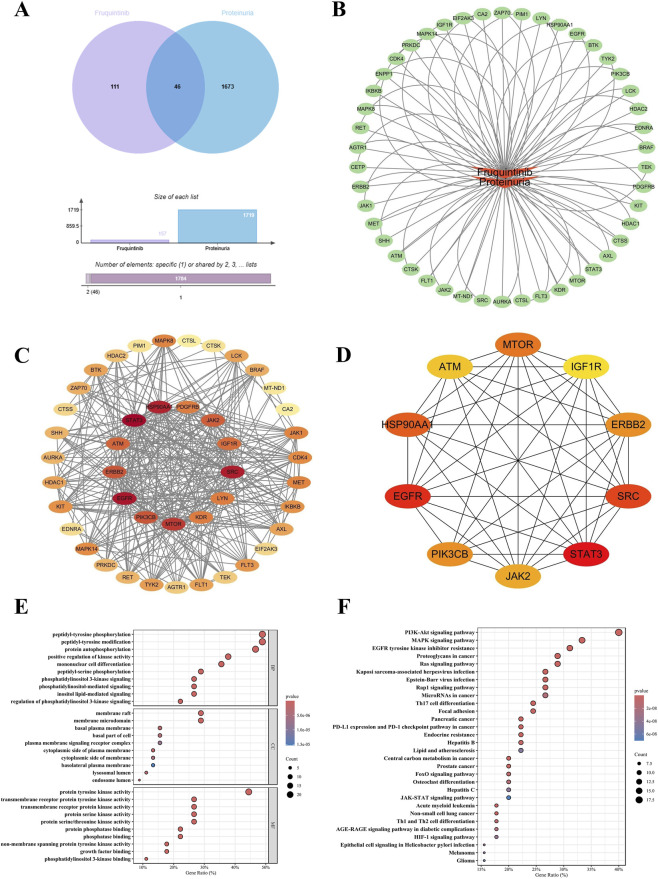
Comprehensive network pharmacology and functional enrichment analysis elucidating the potential molecular mechanisms of Fruquintinib associated proteinuria. **(A)** Venn diagram illustrating the intersection of Fruquintinib putative targets and proteinuria associated targets yielding forty six common targets. **(B)** The drug target disease interaction network constructed utilizing Cytoscape software to map complex regulatory relationships. **(C)** Protein interaction network of the overlapping targets derived from the STRING database. Node color intensity directly correlates with the degree value where darker red indicates a higher frequency of network connectivity. **(D)** The top ten essential hub genes including SRC and STAT3 identified by the CytoHubba plugin based on degree centrality. **(E)** Gene Ontology enrichment analysis of the intersecting targets categorized by biological process, cellular component, and molecular function. **(F)** Kyoto Encyclopedia of Genes and Genomes pathway enrichment analysis highlighting the top twenty statistically significant signaling cascades prominently featuring the PI3K Akt and MAPK pathways.

While the FAERS analysis revealed multiple safety signals, we prioritized proteinuria for mechanistic investigation due to its critically high disproportionality (ROR = 16.92) and clinical significance as a dose-limiting toxicity. Unlike general constitutional symptoms, proteinuria implies specific glomerular filtration barrier injury. The molecular basis of this toxicity likely extends beyond simple VEGF blockade to include complex off-target kinase modulation, necessitating further investigation. We retrieved 157 potential targets for Fruquintinib and 1,719 targets associated with proteinuria. The intersection analysis identified 46 shared targets (Figure 4A), which were mapped into a “Drug-Target-Disease” regulatory network (Figure 4B). To evaluate the functional connectivity, a PPI network was constructed (Figure 4C), where nodes with higher connectivity are displayed in darker red. Topological analysis using CytoHubba identified the top 10 hub genes: SRC, STAT3, EGFR, HSP90AA1, PIK3CB, JAK2, ERBB2, IGF1R, MTOR, and ATM (Figure 4D). The high centrality of SRC and STAT3 suggests that Fruquintinib may disrupt renal function through specific phosphorylation cascades governing podocyte integrity. Functional enrichment analysis provided further biological context. GO annotation (Figure 4E) indicated that the targets are primarily enriched in peptidyl-tyrosine phosphorylation and transmembrane receptor protein tyrosine kinase signaling, with cellular components localized to membrane rafts. KEGG pathway analysis (Figure 4F) highlighted significant enrichment in the PI3K-Akt signaling pathway, MAPK signaling pathway, and EGFR tyrosine kinase inhibitor resistance. These findings suggest that the clinical signal of proteinuria associated with Fruquintinib might be mechanistically linked to the concurrent dysregulation of multiple survival signaling pathways essential for renal cell maintenance.

### 3.7 Molecular validation of core targets

**FIGURE 5:**
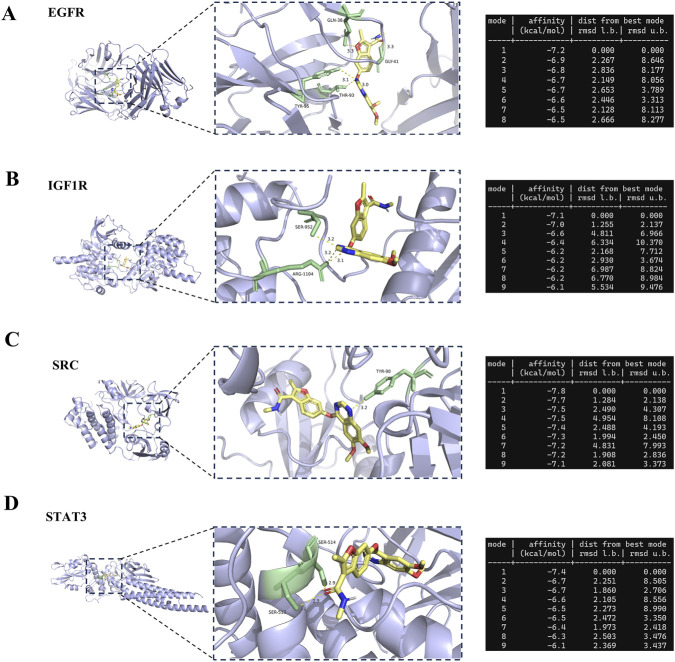
Molecular docking validation and predicted binding conformations of Fruquintinib with prioritized essential hub targets. The three dimensional visualizations display the optimal theoretical binding conformations of Fruquintinib within the active catalytic pockets of **(A)** Epidermal Growth Factor Receptor (EGFR), **(B)** Insulin Like Growth Factor 1 Receptor (IGF1R), **(C)** SRC kinase (SRC), and **(D)** Signal Transducer and Activator of Transcription 3 (STAT3). For each target the left panel shows the overall spatial orientation of the protein and ligand complex while the center panel provides a magnified view detailing the specific interaction interface. Fruquintinib is rendered in yellow sticks and the critical interacting amino acid residues are explicitly labeled in green. Yellow dotted lines indicate intermolecular hydrogen bonds with the corresponding bond lengths explicitly displayed in Angstroms. The tables on the right list the calculated binding affinity in kilocalories per mole and the Root Mean Square Deviation values for the top nine binding modes generated by AutoDock Vina. All top ranked conformational poses consistently exhibited robust binding energies lower than negative seven point zero kilocalories per mole indicating highly favorable theoretical molecular interactions.

To verify the biological plausibility of the network predictions, we prioritized four key signaling kinases—SRC, STAT3, EGFR, and IGF1R—for structural verification. This selection was based on their high degree centrality in the PPI network and their critical roles in renal cell signal transduction. The simulation results demonstrated that Fruquintinib exhibited potent SRC inhibition for all selected targets, with binding energies consistently lower than −7.0 kcal/mol (Figure 5). Specifically, SRC exhibited the strongest binding affinity (−7.8 kcal/mol), forming a stable complex anchored by a hydrogen bond with residue TYR-90 (bond length: 3.2 Å) within the hydrophobic pocket (Figure 5C). STAT3 also showed robust interaction (−7.4 kcal/mol), stabilized by dual hydrogen bonds with SER-513 (2.9 Å) and SER-514 (2.9 Å) (Figure 5D). For EGFR (−7.2 kcal/mol), the ligand fit snugly into the active site, forming a dense hydrogen bond network with GLN-38 (3.3 Å), GLY-41 (3.3 Å), THR-93 (3.1 Å), and TYR-95 (3.0 Å) (Figure 5A). Similarly, IGF1R (−7.1 kcal/mol) interacted with Fruquintinib via residues SER-952 (3.2 Å) and ARG-1104 (3.1 Å) (Figure 5B). These structural data confirm that Fruquintinib can directly occupy the active sites of these targets. The exceptionally high affinity for SRC and STAT3 is particularly noteworthy, as these kinases are pivotal regulators of podocyte cytoskeletal dynamics, offering a molecular explanation for the drug-induced proteinuria observed in the FAERS analysis.

### 3.8 Dynamic stability of fruquintinib with SRC and STAT3

**FIGURE 6:**
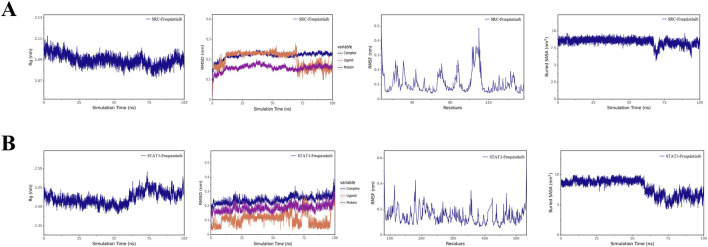
Comprehensive trajectory analysis derived from one hundred nanosecond molecular dynamics simulations evaluating the temporal stability of Fruquintinib complexed with core regulatory targets. Detailed temporal dynamic profiles are presented for the **(A)** SRC Fruquintinib and **(B)** STAT3 Fruquintinib complexes under simulated physiological conditions. The sequential analytical panels display specific structural metrics from left to right. The Radius of Gyration (Rg) measures the overall structural compactness and folding stability of the protein throughout the simulation. The Root Mean Square Deviation (RMSD) quantifies the structural deviation and temporal equilibration of the entire complex indicated by the blue line, the isolated ligand by the orange line, and the protein backbone by the purple line relative to their initial minimized conformations. The Root Mean Square Fluctuation (RMSF) depicts the localized flexibility and dynamic behavior of individual constituent amino acid residues. The Buried Solvent Accessible Surface Area (SASA) represents the extent of the hydrophobic contact interface maintained between the drug molecule and the protein pocket where the stabilization of this area over time strongly indicates persistent and robust ligand binding.

Although molecular docking confirmed static binding potential, we proceeded with MD simulations to verify whether Fruquintinib remains stably bound within the pockets of SRC and STAT3 in a dynamic, solvated environment. These two targets were selected as they exhibited the superior binding scores (<-7.4 kcal/mol) among the tested kinases and are biologically relevant to renal cell integrity. The simulation trajectories over 100 ns indicated that the SRC-Fruquintinib complex achieved remarkable stability (Figure 6A). The Radius of Gyration (Rg) maintained a compact profile around 2.08–2.10 nm, indicating no protein unfolding. The RMSD of the protein–ligand complex (blue line) stabilized within the first 10 ns and remained around 0.22 nm thereafter, indicating a stable binding conformation, suggesting a highly stable binding posture. The RMSF plot (third panel) showed that flexibility was confined to the surface loop regions (residues ∼90-110), while the binding core remained rigid. For the STAT3-Fruquintinib complex (Figure 6B), the system also demonstrated stability, though with distinct dynamic features. The complex RMSD stabilized around 0.25 nm. Notably, the Buried SASA analysis (fourth panel) revealed a significant transition at ∼60 ns, where the contact area dropped from ∼9.0 to ∼6.5 nm^2^ before stabilizing. This likely reflects an “induced-fit” mechanism, where the ligand underwent a conformational adjustment to bury itself deeper into the hydrophobic pocket. Despite this adjustment, the complex remained intact without dissociation, confirming strong affinity.

## 4 Discussion

To our knowledge, this study represents the first comprehensive “bedside-to-bench” investigation of Fruquintinib, integrating real-world pharmacovigilance data with molecular dynamics simulations. While pivotal trials such as FRESCO and FRESCO-2 established the baseline efficacy and safety of Fruquintinib in mCRC, they were conducted on highly selected populations, potentially underestimating the complexity of adverse events in broader clinical practice (Dasari et al., 2023; Li et al., 2018). Our FAERS analysis not only validated known class-related toxicities—such as hypertension and hand–foot skin reaction—but, more importantly, prioritized proteinuria as a critical safety signal with an exceptionally high reporting odds ratio (ROR = 16.92, 95% CI: 11.81–24.23). Unlike subjective symptoms, high-grade proteinuria indicates specific organ injury that directly impacts treatment continuity. Consequently, we employed a reverse translational approach to decipher the molecular drivers of this toxicity.

Proteinuria emerged as the most significant organ-specific signal in our dataset. Mechanistically, the prevailing hypothesis attributes TKI-induced proteinuria solely to the “on-target” inhibition of VEGF signaling in glomerular endothelial cells, which causes microthrombotic injury and loss of fenestrations (Tian et al., 2025; Lian et al., 2026; Eremina et al., 2008). However, this hemodynamic model fails to fully explain the direct podocyte injury often observed in biopsies. A major novelty of our work is the identification of a potential “dual-hit mechanism” driven by off-target kinase modulation.

Through network pharmacology and molecular dynamics simulations, we identified SRC and STAT3 as high-affinity off-target mediators of Fruquintinib. Our docking results revealed that Fruquintinib binds to the active pocket of SRC with a binding energy of −7.8 kcal/mol, surpassing even that of EGFR, and molecular dynamics simulations quantitatively corroborated the structural equilibrium of this interaction, as evidenced by the root mean square deviation of the entire complex consistently plateauing at 0.22 nm over the simulation trajectory. This is biologically profound because SRC kinase is a pivotal regulator of the podocyte slit diaphragm, the final barrier to protein filtration (Verma et al., 2006). Physiologically, SRC phosphorylates nephrin and synaptopodin to maintain cytoskeletal integrity; its inhibition has been experimentally linked to podocyte foot process effacement and massive proteinuria (Yanagida-Asanuma et al., 2007; Jones et al., 2006). This mirrors findings with other multi-kinase inhibitors like dasatinib, where strong SRC structural engagement drives direct podocyte toxicity independent of hemodynamic changes (Wu et al., 2024). Thus, based on our computational models, we postulate that Fruquintinib might contribute to the clinical development of proteinuria through a potential synergistic effect encompassing vascular injury via receptor inhibition combined with direct epithelial destabilization via SRC blockade.

Similarly, the strong interaction identified between Fruquintinib and STAT3 (−7.4 kcal/mol; SASA stabilization at 60 ns) adds another layer of complexity. While STAT3 is a validated oncogenic target in CRC, its basal activity in the kidney is essential for tubulointerstitial homeostasis and repair following injury (O'Shea et al., 2015). The “induced-fit” binding mode observed in our simulation suggests that Fruquintinib may suppress STAT3 signaling, potentially impairing the kidney’s reparative capacity following the initial vascular insult. This multi-target engagement distinguishes Fruquintinib from highly specific monoclonal antibodies and may explain the persistent nature of renal events observed in the FAERS database.

Beyond renal toxicity, our analysis captured a broad spectrum of adverse events, most notably myelosuppression (ROR = 29.31, 95% CI: 23.40–36.71) and dysphonia (ROR = 21.96, 95% CI: 18.37–26.26). Although these signals exhibited higher reporting disproportionality than proteinuria, they were not prioritized for our *in silico* mechanistic pipeline for specific clinical and pharmacological reasons. First, the exceptionally high signal for myelosuppression is heavily confounded by the clinical characteristics of the mCRC population. These patients are typically heavily pretreated and have extensive cumulative exposure to highly myelotoxic systemic chemotherapies (e.g., oxaliplatin, irinotecan, and fluorouracil). Mechanistically, while potent VEGFR blockade by Fruquintinib may compromise VEGF-dependent hematopoietic stem cell niche maintenance in the bone marrow, isolating the drug’s direct molecular off-target toxicity from profound background chemotherapeutic bone marrow exhaustion is extremely challenging in a real-world dataset. Second, as previously noted, dysphonia is widely recognized as a predictable and manageable class effect of anti-angiogenic therapies. It is generally attributable to on-target laryngeal microvascular regression, mucosal ischemia, and localized edema rather than a profound, irreversible structural organ injury (Maitland et al., 2009; Scartozzi et al., 2009). In stark contrast, high-grade proteinuria represents a direct, dose-limiting, and severe structural disruption of the glomerular filtration barrier. Because its etiology often extends beyond simple on-target hemodynamic changes to include direct podocyte toxicity, it served as an optimal and critically necessary candidate for identifying novel molecular off-target mediators (such as SRC and STAT3) through biophysical modeling. Analysis of demographic variations revealed that females were more predisposed to cutaneous and vascular toxicities including hypertension and hand–foot syndrome, while males were at a higher risk of hemorrhagic events. These findings align with sex-based pharmacokinetic differences described for other kinase inhibitors, suggesting that dose optimization based on sex and tolerability could be explored in future studies (Vasudev and Reynolds, 2014; Lambea et al., 2012; Lankheet et al., 2014).

The translation of our computational insights into practical clinical management represents a critical step forward in optimizing patient safety. Recognizing that Fruquintinib associated proteinuria is likely driven by direct structural damage to the podocyte cytoskeleton via SRC and STAT3 inhibition rather than mere hemodynamic shifts, proactive monitoring protocols must be established. Clinicians must conduct a comprehensive baseline assessment of renal function prior to initiating therapy. Given that our temporal analysis identified the first 30 days of treatment as the period of peak adverse event incidence, oncologists must prioritize rigorous urinalysis and mandate frequent renal evaluations during this initial window. For patients exhibiting progressive or high grade proteinuria, the prompt integration of renoprotective therapies, particularly angiotensin converting enzyme inhibitors or angiotensin receptor blockers, is strongly recommended. These pharmacological interventions may provide a dual therapeutic benefit by alleviating intraglomerular hypertension and preserving podocyte structural integrity. Furthermore, severe or persistent renal toxicities necessitate timely multidisciplinary collaboration with nephrologists to carefully weigh the benefits of temporary drug interruption or precise dose adjustments against the risk of compromising oncological efficacy.

While our computational framework provides a compelling theoretical foundation for the observed dual hit renal toxicity, we unequivocally reiterate the absolute necessity for rigorous experimental validation to corroborate these proposed molecular mechanisms. Future research endeavors should be specifically designed to elucidate the downstream functional consequences of Fruquintinib on renal tissue homeostasis. We strongly advocate for targeted *in vitro* kinase activity assays to directly quantify the inhibitory kinetics of Fruquintinib against both SRC and STAT3. Subsequent preclinical investigations utilizing cultured human podocyte cell lines are required to objectively evaluate cytoskeletal dysregulation, nephrin phosphorylation status, and cellular viability following targeted drug exposure. Concurrently, comprehensive *in vivo* studies employing animal models subjected to chronic Fruquintinib exposure are necessary to sequentially monitor the integrity of the glomerular filtration barrier and assess histological alterations. Ultimately, correlating these preclinical findings with real world histopathological evaluations derived from protocol directed clinical renal biopsies will be essential to definitively bridge the conceptual gap between our biophysical predictions and actual patient pathology.

Our study has several limitations inherent to its retrospective and computational design that must be acknowledged. First, as a spontaneous reporting system, the FAERS database is inherently subject to reporting bias, such as underreporting and selective reporting. Furthermore, the database lacks denominator data regarding total drug exposure, which precludes the calculation of true incidence rates for the observed adverse events. Importantly, our pharmacovigilance analysis cannot definitively exclude potential confounding by indication and underlying comorbidities. Given that Fruquintinib is primarily prescribed for patients with heavily pretreated metastatic colorectal cancer, critical confounders such as advanced disease severity, pre existing baseline renal dysfunction, prior exposure to other vascular endothelial growth factor receptor inhibitors, and the extensive use of combination therapies may independently precipitate or exacerbate the observed safety signals. Second, Table 3 shows substantial missing demographic information in FAERS, especially body weight (81.1%) and age (49.6%), with less missingness for sex (7.0%). This reflects an inherent limitation of spontaneous, voluntary reporting with non-mandatory fields. As a result, demographic subgroup analyses have reduced power and limited generalizability, and any age- or sex-stratified safety signals should be interpreted cautiously because complete-case records may not be representative of all patients exposed to fruquintinib. Third, although our molecular docking and 100-ns MD simulations offer rigorous, high-resolution biophysical insights into the drug-target interactions, we must strongly emphasize that these molecular models remain strictly theoretical. Computational predictions of structural binding affinity do not automatically equate to functional enzymatic inhibition *in vivo*. Therefore, the specific off-target inhibition of SRC and STAT3 by Fruquintinib, and its direct translation to podocyte injury, remains a hypothesis-generating finding that unequivocally mandates future experimental validation. Subsequent studies employing *in vitro* kinase activity assays to quantify inhibitory effects, cell-based podocyte functional models, and real-world histopathological evaluations via *in vivo* renal biopsies are absolutely essential to definitively confirm this proposed etiology. Nevertheless, the convergence of strong real-world pharmacovigilance signals and robust structural biology evidence provides potential grounds for this novel mechanistic hypothesis and points to a potential direction for future molecular toxicology research.

## 5 Conclusion

In summary, this study delineates the real world adverse event associations of Fruquintinib and proposes a novel computational molecular hypothesis regarding its potential renal toxicity. We highlight proteinuria as a critical safety signal that may be mechanistically linked to both on target receptor blockade and the potential off target inhibition of SRC and STAT3. These findings support a paradigm shift towards precision pharmacovigilance, emphasizing early protection of podocyte function in mCRC patients.

## Data Availability

The original contributions presented in the study are included in the article/Supplementary Material, further inquiries can be directed to the corresponding authors.
