# Supplementary material for: Real-world pharmacovigilance and molecular mechanisms of fruquintinib: SRC and STAT3 as potential off-target mediators of proteinuria
Source: Front Pharmacol. 2026 Mar 13;17:1796154. doi: 10.3389/fphar.2026.1796154 (PMC13021642; doi:10.3389/fphar.2026.1796154)
Supplement: Supplementary file 1 [file Supplementaryfile1.docx]

Supplementary Material

# Supplementary Figures and Tables

## Supplementary Figures


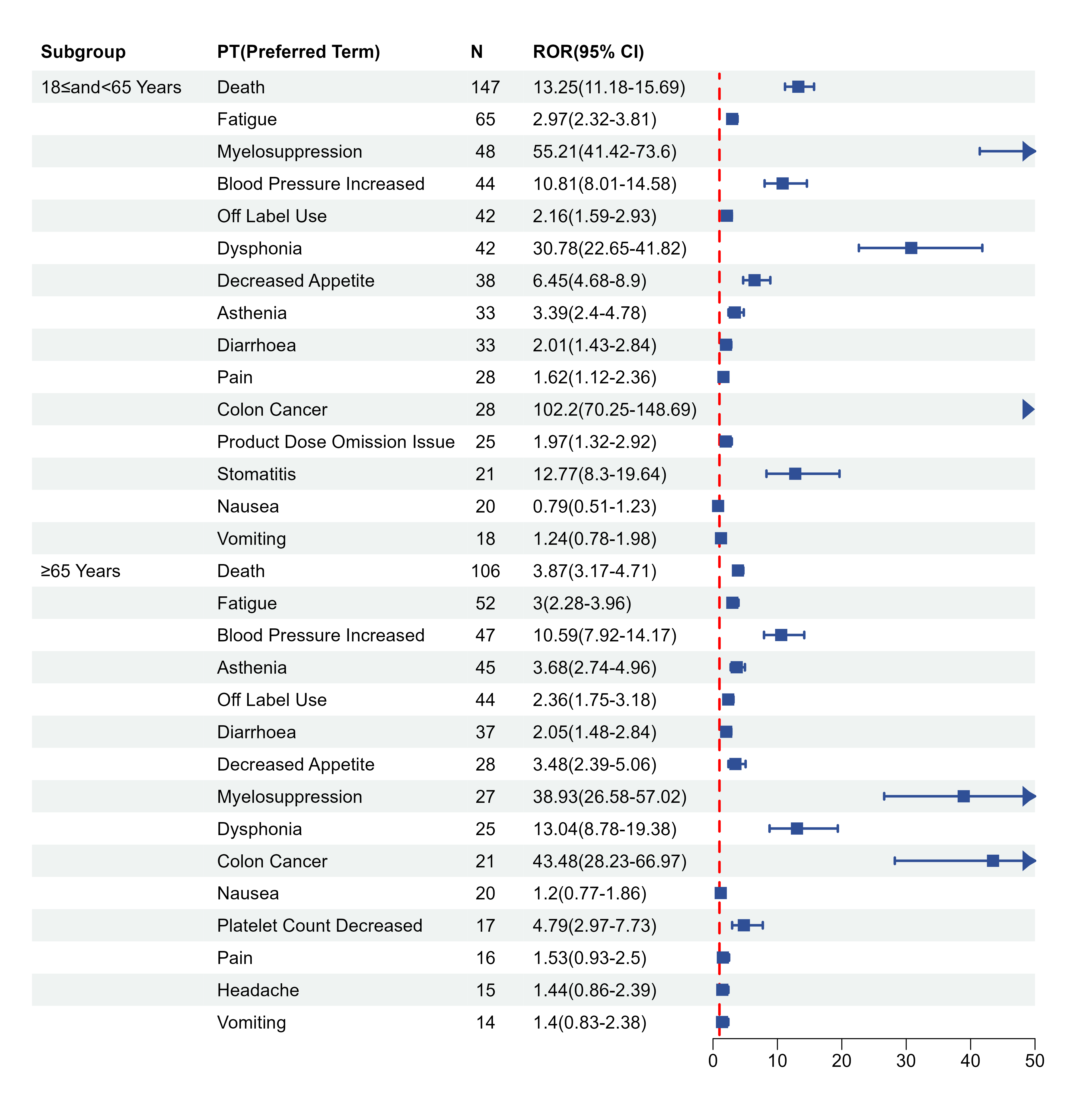


**Supplementary Figure 1.** **Forest plots of age-specific reporting odds ratios (RORs) for preferred terms -level adverse events associated with Fruquintinib.**


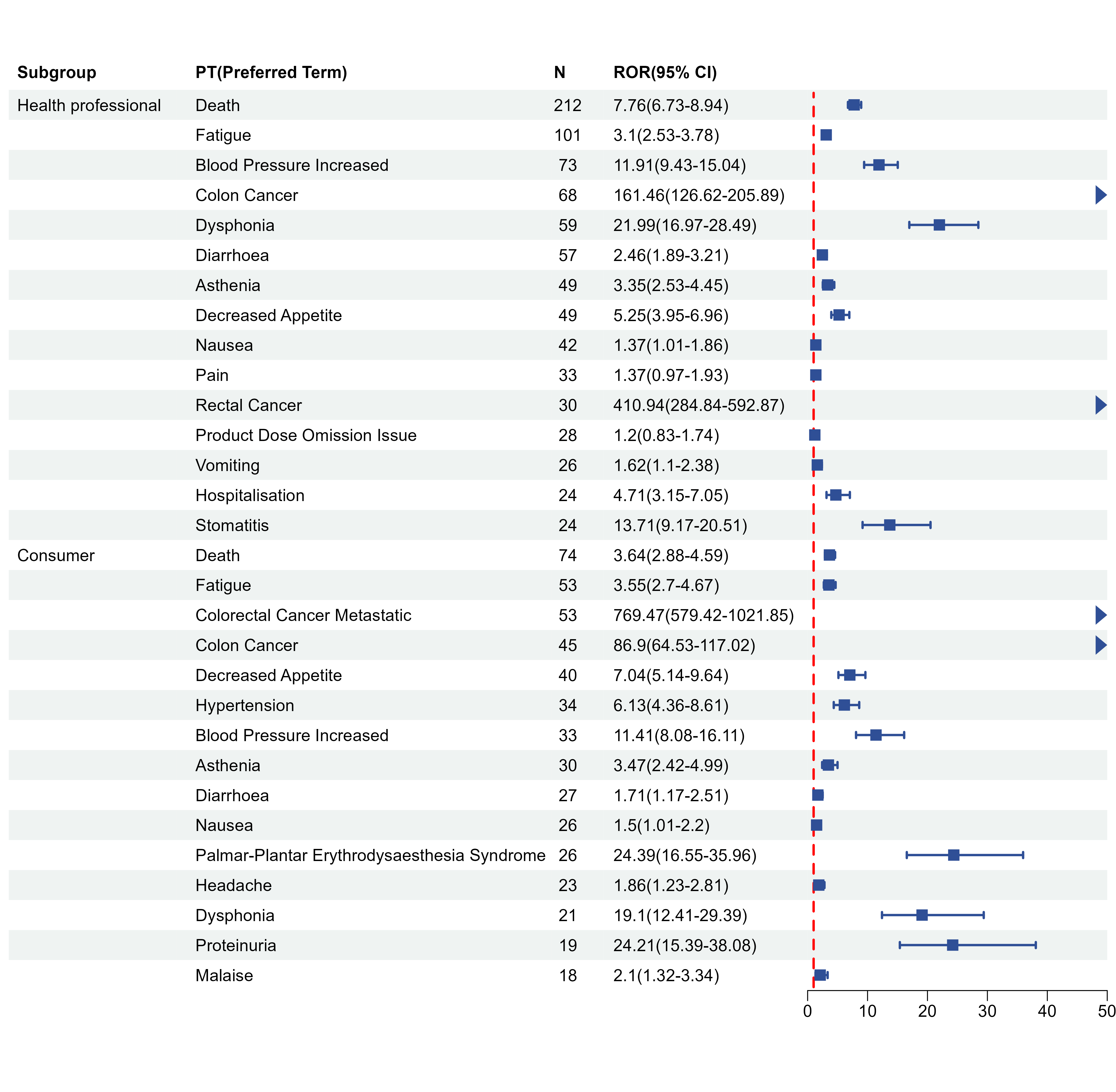


**Supplementary Figure 2.** **Forest plots of reporter-type–specific reporting odds ratios (RORs) for preferred terms -level adverse events associated with Fruquintinib.**

## Supplementary Table

| **SOC** | **PT** | **N** | **ROR (95%Cl)** | **IC (IC025)** | **PRR (95%Cl)** | **EBGM (EBGM05)** |
| --- | --- | --- | --- | --- | --- | --- |
| Blood and lymphatic system disorders | Myelosuppression | 77 | 29.31 (23.4-36.71) | 4.85 (4.08) | 28.95(2072.25) | 28.86(23.04) |
| Endocrine disorders | Hypothyroidism | 19 | 6.22(3.96-9.76) | 2.63(1.65) | 6.2(82.93) | 6.2(3.95) |
| Eye disorders | Eye ulcer | 1 | 19.1(2.68-135.88) | 4.25(-1.12) | 19.1(17.11) | 19.06(2.68) |
|  | Scleral discolouration | 1 | 15.1(2.12-107.35) | 3.91(-1.14) | 15.09(13.14) | 15.07(2.12) |
| Gastrointestinal disorders | Diarrhoea | 139 | 2.29(1.94-2.71) | 1.18(0.92) | 2.26(98.79) | 2.26(1.91) |
|  | Nausea | 95 | 1.25(1.02-1.53) | 0.32(0.02) | 1.25(4.68) | 1.25(1.02) |
|  | Vomiting | 60 | 1.33(1.03-1.72) | 0.41(0.03) | 1.33(4.96) | 1.33(1.03) |
|  | Stomatitis | 56 | 9.59(7.3-12.47) | 3.25(2.66) | 9.51(426.18) | 9.5(7.3) |
|  | Abdominal pain upper | 52 | 2.65(2.02-3.48) | 1.4(0.95) | 2.63(52.88) | 2.63(2) |
| General disorders and administration site conditions | Death | 438 | 5.62(5.1-6.19) | 2.4(2.24) | 5.28(1540.45) | 5.28(4.79) |
|  | Fatigue | 248 | 3.4(3-3.87) | 1.72(1.52) | 3.31(403.74) | 3.3(2.91) |
|  | Asthenia | 130 | 3.6(3.03-4.29) | 1.83(1.54) | 3.55(239.2) | 3.55(2.98) |
|  | Pain | 83 | 1.37(1.11-1.71) | 0.45(0.13) | 1.37(8.36) | 1.37(1.1) |
|  | Illness | 31 | 3.72(2.61-5.29) | 1.89(1.26) | 3.7(61.22) | 3.7(2.6) |
| Hepatobiliary disorders | Liver disorder | 14 | 3.27(1.94-5.52) | 1.71(0.76) | 3.26(22) | 3.26(1.93) |
|  | Hepatic function abnormal | 8 | 2.26(1.13-4.53) | 1.18(0.02) | 2.26(5.62) | 2.26(1.13) |
|  | Jaundice | 7 | 2.6(1.24-5.46) | 1.38(0.09) | 2.6(6.9) | 2.6(1.24) |
|  | Hepatic failure | 7 | 2.36(1.12-4.95) | 1.24(-0.01) | 2.36(5.46) | 2.36(1.12) |
|  | Biliary obstruction | 6 | 20.01(8.98-44.6) | 4.32(1.34) | 19.99(108.03) | 19.95(8.95) |
| Infections and infestations | Urinary tract infection | 29 | 1.77(1.23-2.55) | 0.82(0.26) | 1.77(9.7) | 1.77(1.23) |
|  | Kidney infection | 6 | 3.16(1.42-7.03) | 1.66(0.18) | 3.15(8.83) | 3.15(1.42) |
|  | Anal abscess | 5 | 9.67(4.02-23.24) | 3.27(0.8) | 9.66(38.78) | 9.65(4.01) |
|  | Respiratory syncytial virus infection | 3 | 3.89(1.25-12.08) | 1.96(-0.27) | 3.89(6.44) | 3.89(1.25) |
|  | Groin abscess | 2 | 17.8(4.45-71.3) | 4.15(-0.24) | 17.8(31.65) | 17.77(4.44) |
| Injury, poisoning and procedural complications | Product dose omission issue | 67 | 1.41(1.11-1.8) | 0.49(0.13) | 1.41(7.94) | 1.41(1.11) |
|  | Stoma site haemorrhage | 6 | 41.57(18.63-92.72) | 5.37(1.52) | 41.52(236.26) | 41.35(18.54) |
|  | Oralcontusion | 1 | 46.71(6.55-333.25) | 5.54(-1.08) | 46.7(44.5) | 46.47(6.51) |
|  | Stoma prolapse | 1 | 99.31(13.84-712.45) | 6.62(-1.08) | 99.3(96.29) | 98.27(13.7) |
|  | Frostbite | 1 | 34.94(4.9-249.01) | 5.12(-1.09) | 34.94(32.84) | 34.81(4.89) |
| Investigations | Blood pressure increased | 173 | 11.86(10.19-13.79) | 3.53(3.22) | 11.54(1668.32) | 11.53(9.91) |
|  | Weight decreased | 52 | 1.92(1.46-2.52) | 0.94(0.51) | 1.91(22.74) | 1.91(1.46) |
|  | Platelet count decreased | 38 | 3.67(2.67-5.05) | 1.87(1.31) | 3.65(73.32) | 3.65(2.65) |
|  | White blood cell count decreased | 23 | 2.17(1.44-3.27) | 1.12(0.46) | 2.17(14.52) | 2.17(1.44) |
|  | Blood bilirubin increased | 15 | 5.6(3.37-9.29) | 2.48(1.39) | 5.58(56.45) | 5.58(3.36) |
| Metabolism and nutrition disorders | Decreased appetite | 127 | 5.49(4.6-6.54) | 2.43(2.12) | 5.39(455.68) | 5.39(4.52) |
|  | Dehydration | 39 | 2.99(2.18-4.1) | 1.57(1.05) | 2.98(51.38) | 2.98(2.17) |
|  | Hypophagia | 16 | 5.85(3.58-9.55) | 2.54(1.48) | 5.83(64.08) | 5.83(3.57) |
|  | Feeding disorder | 6 | 3.37(1.51-7.5) | 1.75(0.24) | 3.37(9.98) | 3.37(1.51) |
| Musculoskeletal and connective tissue disorders | Pain in extremity | 51 | 1.76(1.33-2.31) | 0.81(0.38) | 1.75(16.43) | 1.75(1.33) |
|  | Backpain | 44 | 1.94(1.44-2.61) | 0.95(0.49) | 1.93(19.83) | 1.93(1.44) |
|  | Sacral pain | 1 | 20.16(2.83-143.44) | 4.33(-1.12) | 20.16(18.17) | 20.12(2.83) |
|  | Mandibular mass | 1 | 62.48(8.74-446.53) | 5.96(-1.08) | 62.47(60.09) | 62.07(8.68) |
|  | Trigger points | 1 | 154.67(21.44-1115.91) | 7.25(-1.08) | 154.64(150.18) | 152.16(21.09) |
| Neoplasms benign, malignant and unspecified | Colon cancer | 162 | 114.24(97.64-133.66) | 6.78(5.81) | 111.2(17489.25) | 109.91(93.94) |
|  | Colorectal cancer metastatic | 83 | 523.31(418.96-653.66) | 8.93(5.84) | 516.11(40459.13) | 489.39(391.8) |
|  | Rectal cancer | 58 | 196.52(151.34-255.19) | 7.58(5.12) | 194.63(10947.77) | 190.72(146.87) |
|  | Metastases to lung | 22 | 20.12(13.23-30.59) | 4.32(2.85) | 20.05(397.36) | 20.01(13.16) |
|  | Metastases to liver | 20 | 11.99(7.73-18.6) | 3.58(2.34) | 11.95(200.56) | 11.94(7.7) |
| Nervous system disorders | Neuropathy peripheral | 44 | 4.87(3.62-6.55) | 2.27(1.72) | 4.84(134.15) | 4.84(3.6) |
|  | Hypersomnia | 11 | 3.97(2.2-7.17) | 1.99(0.83) | 3.96(24.36) | 3.96(2.19) |
|  | Speech disorder | 10 | 1.94(1.04-3.6) | 0.95(-0.04) | 1.94(4.53) | 1.94(1.04) |
|  | Taste disorder | 9 | 5.12(2.66-9.85) | 2.35(0.94) | 5.12(29.81) | 5.12(2.66) |
|  | Brain fog | 9 | 9.91(5.15-19.07) | 3.31(1.47) | 9.9(71.94) | 9.89(5.14) |
| Product issues | Product physical issue | 5 | 2.61(1.08-6.27) | 1.38(-0.14) | 2.61(4.95) | 2.61(1.08) |
| Psychiatric disorders | Grief reaction | 1 | 17.57(2.47-124.98) | 4.13(-1.12) | 17.57(15.59) | 17.54(2.47) |
| Renal and urinary disorders | Proteinuria | 30 | 16.92(11.81-24.23) | 4.07(2.96) | 16.84(446.28) | 16.81(11.74) |
|  | Renal impairment | 24 | 2.99(2-4.47) | 1.58(0.89) | 2.98(31.69) | 2.98(2) |
|  | Nephrotic syndrome | 12 | 18.13(10.29-31.97) | 4.18(2.16) | 18.1(193.51) | 18.07(10.25) |
|  | Chromaturia | 7 | 3.08(1.47-6.46) | 1.62(0.27) | 3.07(9.8) | 3.07(1.46) |
|  | Renal-limited thrombotic microangiopathy | 3 | 209.73(66.78-658.65) | 7.68(0.52) | 209.62(609.35) | 205.09(65.3) |
| Reproductive system and breast disorders | Nipple pain | 2 | 10.1(2.52-40.44) | 3.33(-0.34) | 10.1(16.38) | 10.09(2.52) |
|  | Pelvic fluid collection | 1 | 14.88(2.09-105.83) | 3.89(-1.14) | 14.88(12.93) | 14.86(2.09) |
|  | Cervix haemorrhage uterine | 1 | 54.22(7.59-387.17) | 5.75(-1.08) | 54.21(51.93) | 53.91(7.55) |
| Respiratory, thoracic and mediastinal disorders | Dysphonia | 123 | 21.96(18.37-26.26) | 4.43(3.94) | 21.53(2404.98) | 21.49(17.97) |
|  | Oropharyngeal pain | 22 | 2.45(1.61-3.73) | 1.29(0.6) | 2.45(18.85) | 2.45(1.61) |
|  | Epistaxis | 21 | 2.87(1.87-4.41) | 1.52(0.78) | 2.87(25.52) | 2.86(1.87) |
|  | Aphonia | 17 | 12.73(7.91-20.5) | 3.66(2.26) | 12.7(182.98) | 12.68(7.88) |
|  | Pulmonary haemorrhage | 3 | 3.82(1.23-11.85) | 1.93(-0.28) | 3.82(6.24) | 3.82(1.23) |
| Skin and subcutaneous tissue disorders | Palmar-plantar erythrodysaesthesia syndrome | 51 | 22.43(17.02-29.55) | 4.47(3.58) | 22.25(1032.78) | 22.2(16.84) |
|  | Dry skin | 32 | 2.6(1.84-3.68) | 1.37(0.8) | 2.59(31.31) | 2.59(1.83) |
|  | Blister | 27 | 5.03(3.45-7.35) | 2.33(1.59) | 5.02(86.87) | 5.01(3.44) |
|  | Skin exfoliation | 17 | 2.16(1.34-3.48) | 1.11(0.34) | 2.16(10.56) | 2.16(1.34) |
|  | Skin ulcer | 8 | 3.17(1.58-6.34) | 1.66(0.39) | 3.17(11.86) | 3.17(1.58) |
| Social circumstances | Sitting disability | 1 | 18.94(2.66-134.78) | 4.24(-1.12) | 18.94(16.96) | 18.91(2.66) |
| Surgical and medical procedures | Hospitalisation | 33 | 2.32(1.65-3.27) | 1.21(0.66) | 2.32(24.76) | 2.32(1.65) |
|  | Colostomy | 2 | 10.85(2.71-43.41) | 3.44(-0.33) | 10.84(17.85) | 10.83(2.71) |
|  | Biliary tract operation | 1 | 131.04(18.21-943.13) | 7.01(-1.08) | 131.01(127.26) | 129.23(17.96) |
|  | Radioembolisation | 1 | 349.43(47.47-2571.98) | 8.4(-1.11) | 349.37(334.97) | 336.93(45.78) |
|  | Abdominal cavity drainage | 1 | 23.07(3.24-164.18) | 4.52(-1.11) | 23.06(21.06) | 23.01(3.23) |
| Vascular disorders | Hypertension | 53 | 2.58(1.97-3.38) | 1.36(0.92) | 2.56(50.73) | 2.56(1.96) |
|  | Malignant hypertension | 2 | 22.79(5.69-91.31) | 4.51(-0.21) | 22.79(41.56) | 22.73(5.67) |
|  | Micro embolism | 1 | 89.85(12.54-643.97) | 6.48(-1.08) | 89.84(87.02) | 89(12.42) |
|  | Varicose vein ruptured | 1 | 31.66(4.44-225.54) | 4.98(-1.09) | 31.65(29.59) | 31.55(4.43) |

SOC, System Organ Class; PT, Preferred Term; N, Number of cases; CI, Confidence Interval; ROR, Reporting Odds Ratio; IC (IC025), Information Component with lower 95% credibility interval; PRR, Proportional Reporting Ratio; EBGM (EBGM05), Empirical Bayes Geometric Mean with lower 95% one-sided confidence bound;

**Supplementary Table 1.** **Disproportionality analysis results of Fruquintinib-associated adverse events based on multiple signal detection methods.**
